# Factors Associated With Poor One‐Month Glasgow Outcome Scale Scores After Traumatic Brain Injury With Intracranial Hemorrhage in Adult Patients Presenting to the Emergency Department

**DOI:** 10.1155/emmi/1023217

**Published:** 2026-05-16

**Authors:** Welawat Tienpratarn, Phichayut Phinyo, Chaiyaporn Yuksen, Sirote Wongwaisayawan, Jiraporn Khorana, Jayanton Patumanond, Suteenun Seesuklom

**Affiliations:** ^1^ Department of Emergency Medicine, Faculty of Medicine, Ramathibodi Hospital, Mahidol University, Ratchathewi, 10400, Bangkok, Thailand, mahidol.ac.th; ^2^ Department of Biomedical Informatics and Clinical Epidemiology (BioCE), Faculty of Medicine, Chiang Mai University, Chiang Mai, 50200, Thailand, cmu.ac.th; ^3^ Center for Clinical Epidemiology and Clinical Statistics, Faculty of Medicine, Chiang Mai University, Chiang Mai, 50200, Thailand, cmu.ac.th; ^4^ Emergency Radiology Unit, Department of Diagnostic and Therapeutic Radiology, Faculty of Medicine, Ramathibodi Hospital, Mahidol University, Ratchathewi, 10400, Bangkok, Thailand, mahidol.ac.th; ^5^ Department of Surgery, Faculty of Medicine, Chiang Mai University, Chiang Mai, 50200, Thailand, cmu.ac.th

**Keywords:** brain injury, glasgow outcome scale, prognosis, traumatic

## Abstract

**Background:**

Traumatic brain injury (TBI) is a major public health concern in Thailand, contributing to substantial morbidity and mortality. This study aimed to identify prognostic factors associated with poor Glasgow Outcome Scale (GOS) scores 1 month after TBI with intracranial hemorrhage (ICH). This is particularly relevant in the emergency department (ED), where early decisions regarding triage, monitoring, and disposition must be made rapidly.

**Methods:**

We conducted a retrospective cohort study at Ramathibodi Hospital, Bangkok, Thailand, including trauma patients aged ≥ 15 years with TBI and ICH who presented to the ED between 2020 and 2022. Outcomes were categorized into three groups based on the 1‐month GOS: unfavorable (GOS 1–2), intermediate (GOS 3–4), and favorable (GOS 5). Clinical factors and CT findings were analyzed using multivariable ordinal logistic regression to identify factors associated with poor GOS scores across these groups.

**Results:**

A total of 227 patients were included in the study. Among them, 31 patients (13.6%) were in the unfavorable group, 81 patients (35.7%) in the intermediate group, and 115 patients (50.7%) in the favorable group. Factors associated with poorer outcomes included elderly patients (age ≥ 65 years) (multivariable odds ratio [mOR] 5.25, 95% confidence interval [CI] 2.33–11.85), low initial systolic blood pressure (SBP < 100 mmHg) (mOR 4.38, 95% CI 1.02–18.86), and initial glasgow coma scale (GCS) scores: severe vs. mild (mOR 49.88, 95% CI 14.26–174.44) and moderate vs. mild (mOR 12.26, 95% CI 3.86–38.98). Other factors included slight pupillary reaction (mOR 8.36, 95% CI 1.76–39.67), although this finding should be interpreted cautiously due to the small number of abnormal observations and wide CIs, as well as subdural hematoma (SDH) (mOR 3.10, 95% CI 1.53–6.25) and midline shift or brain herniation (mOR 4.41, 95% CI 1.84–10.57).

**Conclusions:**

These factors were associated with poorer 1‐month GOS scores and may support early risk stratification in adult TBI patients with traumatic ICH.

## 1. Introduction

Traumatic brain injury (TBI) is a leading cause of morbidity and mortality, particularly in low‐ and middle‐income countries [1–3]. TBI accounts for a substantial proportion of trauma‐related mortality in Thailand [4, 5]. Intracranial hemorrhage (ICH), a common complication, significantly impacts neurological outcomes, underscoring the importance of early prognostic assessment in emergency settings.

Several studies have identified clinical and radiological prognostic factors in TBI, including age, Glasgow Outcome Scale (GCS) score, pupillary reactivity, extracranial injury, and CT abnormalities. However, much of the literature has focused on mortality or longer‐term outcomes, such as three‐ or 6‐month functional recovery, whereas data on early 1‐month functional outcome in patients with traumatic ICH remain limited [6–9].

The Glasgow Outcome Scale (GOS) is a widely used measure of functional recovery after TBI and was selected in this study to assess early outcome at 1 month.

Early prognostic assessment in the emergency department (ED) is essential for several aspects of patient care, including initial triage prioritization, decisions regarding admission to the general ward or intensive care unit (ICU), determination of monitoring intensity, and early communication with patients’ families regarding expected short‐term outcomes. Identifying factors associated with early functional outcomes may therefore support risk stratification and guide initial management decisions in the ED setting.

Therefore, this study aimed to identify factors associated with 1‐month neurological outcomes in adult patients with TBI and traumatic ICH. This study was conducted in a high‐volume university‐based trauma center that manages more than 3500 trauma visits annually, making it a relevant setting for evaluating early prognostic factors in TBI.

## 2. Methods

### 2.1. Study Design, Participants, and Setting

This single‐center retrospective cohort study was conducted at Ramathibodi Hospital, a university‐based Level II trauma center in metropolitan Thailand. We included consecutive trauma patients aged 15 years or older with TBI and ICH identified on CT between January 2020 and December 2022. In our clinical setting, patients aged ≥ 15 years are managed within adult trauma care pathways and were therefore considered representative of adult patients.

This study received ethical approval from the Committee on Human Rights Related to Research Involving Human Subjects at Ramathibodi Hospital (IRB COA. MURA2023/728). Due to its retrospective design, the need for informed consent was waived.

This study was conducted and reported in accordance with the Strengthening the Reporting of Observational Studies in Epidemiology (STROBE) guidelines.

### 2.2. Data Gathering

We collected data from electronic medical records (EMR) involving general variables about sex, age [9–12], elderly age defined as age ≥ 65 years [11, 13], type of escort (self‐escort or ambulance), ESI triage level [1–3], type of injury (blunt or penetrating), mechanism of injury (high and low mechanism), injury severity score (ISS), high ISS defined as ISS ≥ 16 [14], neurodegenerative disease (Alzheimer’s disease [15] or Parkinson disease [16]), old cerebrovascular accidents [17], medications (antiplatelets and anticoagulants) [18], syncope or dizziness prior head injury [19], and previous TBI with ICH. High‐mechanism injuries included road traffic accidents (motorcycle accidents, vehicle collisions, and pedestrian injuries), falls from height (fall > 6 m) [20], and others (pedestrian crash by train). Low‐mechanism injuries included falling on the ground.

Initial clinical observation consisted of vital signs and physical examination. Vital signs assessed included systolic blood pressure (SBP), with hypotension defined as SBP < 100 mmHg [21]; heart rate (HR); respiratory rate (RR); and oxygen saturation (SpO_2_), with hypoxia defined as SpO_2_ < 95 [20]. Additional factors included GCS score and pupillary reactivity [9, 22, 23]. Major extracranial injuries were classified as injuries requiring hospital admission, simplifying data collection and minimizing redundancy in prognostic prediction [9].

Traumatic ICH was defined as any acute traumatic hemorrhagic lesion identified on the initial CT scan or formal radiology report, including epidural hematoma, subdural hematoma (SDH), traumatic subarachnoid hemorrhage, intraventricular hemorrhage, petechial hemorrhage (small punctate intraparenchymal lesions), and intraparenchymal hemorrhage (focal hematoma or hemorrhagic contusion). Multiple lesion types could coexist in the same patient. Midline shift or brain herniation was recorded as a single composite variable representing radiological evidence of mass effect.

Disposition type included discharge home and follow‐up, general ward or ICU admission, and dead at ED. The neurosurgical intervention included emergency craniotomy or craniectomy with clot removal.

Patients with missing 1‐month GOS were excluded from the primary analysis. Five patients with missing initial hemodynamic variables were also excluded because these variables were prespecified candidate predictors. No multiple imputation was performed, and a complete‐case analysis approach was used.

A complete‐case analysis approach was chosen because the proportion of missing data was small and involved key predictor variables. We did not perform multiple imputation because the limited number of missing cases was unlikely to substantially influence the overall results, and assumptions required for imputation could not be reliably verified.

### 2.3. Outcome Measurements

The GOS is a widely used and reliable tool for assessing neurological recovery and functional outcomes in patients with TBI. Measuring the GOS at 1 month post‐TBI is a well‐established practice, providing early prognostication during the recovery phase, evaluating the effectiveness of acute treatment (including interventions in the ED), and serving as a standard outcome measurement [24–27]. In this study, the outcome measure was the 1‐month GOS score, which was assessed and recorded by physicians in various settings: the outpatient clinic for discharged patients, the inpatient ward or ICU for hospitalized patients, or the ED for patients who died.

The 1‐month GOS should be interpreted as an early functional outcome rather than a measure of definitive long‐term recovery. The choice of a 1‐month outcome reflects the need for early prognostication in emergency medicine, including short‐term care planning, discharge decisions, follow‐up organization, and early rehabilitation considerations.

We categorized the GOS scores into five levels, ranging from 1 (worst) to 5 (best), assessed 1 month after TBI with ICH. GOS one indicated death or nonsurvival; GOS 2 represented a persistent vegetative state, defined as being in a coma or unresponsive and speechless for weeks or months (severe neurological deficit). GOS 3 was severe disability, characterized by dependence on daily support for basic needs (significant neurological deficit). GOS four indicated moderate disability, defined as some impairment but independence in daily life at home, while being dependent on assistance outside the home. Finally, GOS 5 represented good recovery, defined as the resumption of normal life and the ability to work, although minor neurological deficits might persist.

The GOS scores were reclassified into three groups to reflect recovery trajectories: unfavorable (GOS 1–2), intermediate (GOS 3–4), and favorable (GOS 5). The unfavorable group included death or vegetative state (GOS 1–2), the intermediate group included severe to moderate disability (GOS 3–4), and the favorable group indicated good recovery (GOS 5). We believe that this categorization is more clinically meaningful and also benefits from balancing the size of the strata, thereby improving statistical efficiency.

GOS was assigned by treating physicians based on routine clinical assessment in the respective care setting. No separate study‐specific standardized assessor training was implemented due to the retrospective design.

### 2.4. Sample Size Estimation

The sample size estimation was based on the distribution of a clinically important predictor from pilot data and was intended to provide a pragmatic estimate of feasibility. However, this calculation was not specifically derived for the final multivariable ordinal regression model and should therefore be interpreted as approximate.

The sample size was estimated using data from an unpublished pilot report conducted between January and December 2022. We performed a sample size calculation to determine the minimal sample size needed to identify significant differences in the predictor distribution—specifically, the presence of midline shift—across the three‐category outcome of GOS scores (with two possible ordinal cut points). This calculation was based on a two‐sample proportions test with a significance level of 0.05% and 80% statistical power. According to the pilot data, the proportions of GOS groups were 52.0% favorable, 37.0% intermediate, and 11.0% unfavorable. A total sample size of 171 patients was deemed sufficient (95 patients in the favorable group, 57 in the intermediate group, and 19 in the unfavorable group).

### 2.5. Statistical Analysis

Statistical analyses were performed using Stata Version 17.0 (StataCorp LLC, College Station, TX, USA), with significance defined as a two‐tailed *p* value < 0.05. Data distribution was assessed using histograms and plots. Categorical data were reported as counts and percentages, while continuous data were presented as mean (SD) for normally distributed data and median (IQR) for nonnormally distributed data. Variables across the three GOS score groups were compared using tests for trends across ordered groups. Multivariable ordinal logistic regression was employed to identify factors associated with poor 1‐month GOS scores.

Candidate variables for the multivariable ordinal logistic regression model were selected based on clinical relevance and univariable association with outcome. Variables considered clinically important or showing a univariable association at *p* < 0.10 were entered into the full model, after which a reduced final model was developed based on statistical significance and clinical interpretability.

The proportional odds assumption was assessed using the Brant test and was not materially violated.

## 3. Results

**FIGURE 1:**
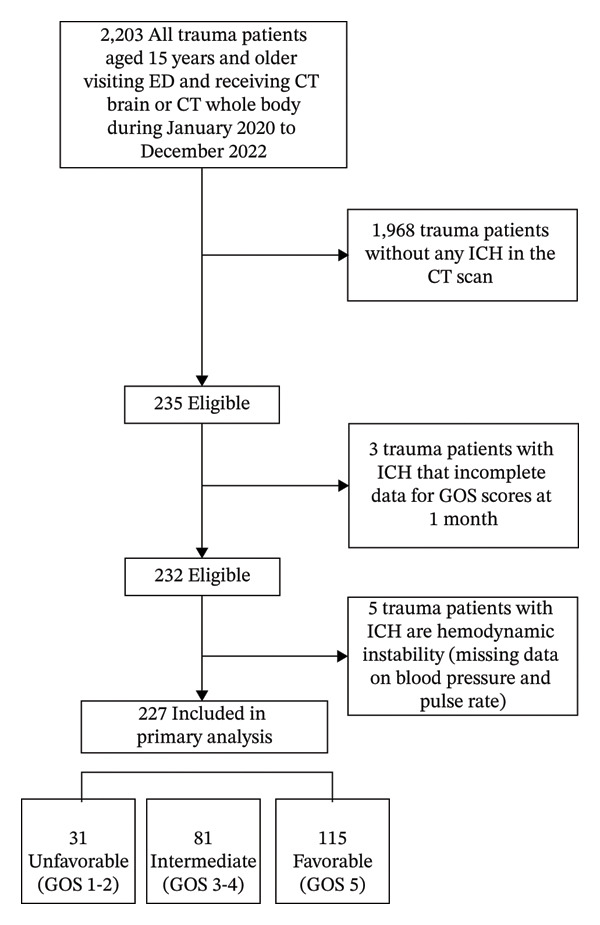
A study flow of 227 TBI patients with ICH were enrolled in the complete retrospective cohort study.

Between January 2020 and December 2022, a total of 2203 adult trauma patients aged 15 years and older underwent brain CT or whole‐body CT scans. Among them, 1968 patients without ICH on CT and 3 patients with ICH who were referred to another hospital and could not be followed up for GOS scores were excluded. Additionally, 5 patients with missing data on blood pressure and HR were excluded. Ultimately, 227 patients met the study criteria and were included in the analysis. The study flow is illustrated in Figure 1.

Of the included patients, 31 (13.6%) were classified in the unfavorable group, 81 (35.7%) in the intermediate group, and 115 (50.7%) in the favorable group.

Most patients had mild TBI at presentation (181/227, 79.7%), whereas 19 (8.4%) had moderate TBI and 27 (11.9%) had severe TBI. This distribution indicates that the cohort was predominantly composed of mild TBI with traumatic ICH, which should be considered when interpreting subsequent prognostic associations. Baseline characteristics of the study population are summarized in the following.

### 3.1. Baseline Characteristics of Adult TBI Patients With ICH

The mean age of the patients was 67.9 ± 21.5 years, with 55.7% being male. Patients aged ≥ 65 years were more common in the unfavorable and intermediate groups. All injuries were blunt trauma, with the majority caused by falls (74.0%). A significant trend of higher ISS was observed in the unfavorable group compared to the intermediate and favorable groups (*p* < 0.001) (Table 1).

**TABLE 1:** Baseline general characteristics of adult TBI patients with ICH classified by 3 groups of GOS scores (unfavorable; GOS 1‐2, intermediate; GOS 3‐4, and favorable; GOS 5).

Variables	All *N* = 227	GOS 1–2 31 (13.6%)	GOS 3–4 81 (35.7%)	GOS 5 115 (50.7%)	*p* for trend
Sex (Male)	127 (55.7)	18 (58.1)	45 (55.6)	64 (55.7)	0.950
Age (years) mean ± SD	67.9 ± 21.5	61.4 ± 26.8	72.3 ± 20.4	66.7 ± 20.2	0.804
Age ≥ 65 years	152 (67.0)	16 (51.6)	62 (76.5)	74 (64.4)	0.728
Type of escort					
‐ Self‐escort	168 (74.0)	15 (48.4)	60 (74.1)	93 (80.9)	< 0.001
‐ Ambulance	59 (26.0)	16 (51.6)	21 (25.9)	22 (19.1)	
ESI triage level					
‐ Level 1	43 (19.0)	17 (54.8)	19 (23.5)	7 (6.1)	< 0.001
‐ Level 2	102 (44.9)	10 (32.3)	35 (43.2)	57 (49.6)	
‐ Level 3	82 (36.1)	4 (12.9)	27 (33.3)	51 (44.3)	
Type of injury					
‐ Blunt	227 (100)	227 31 (13.6%)	81 (35.7%)	115 (50.7%)	N/A
‐ Penetrating	0 (0)	0 (0)	0 (0)	0 (0)	
Mechanism of injury					
Low energy mechanism (Falling)	168 (74.0)	17 (54.8)	60 (74.1)	91 (79.1)	0.012
High energy mechanism	59 (26.0)	14 (45.2)	21 (25.9)	24 (20.9)	
‐ Road traffic accident	42 (18.5)	9 (29.0)	16 (19.8)	17 (14.8)	
‐ Fall from height	12 (5.3)	2 (6.5)	5 (6.2)	5 (4.4)	
‐ Other	5 (2.2)	3 (9.7)	0 (0)	2 (1.7)	
Injury severity score (ISS) mean (IQR)	8 (5, 12)	18 (10, 25)	8 (5, 12)	5 (5, 9)	< 0.001
High ISS (ISS ≥ 16)	40 (17.6)	20 (64.5)	16 (19.8)	4 (3.5)	< 0.001
Underlying diseases					
‐ Neurodegenerative	21 (9.3)	2 (6.5)	13 (16.1)	6 (5.2)	0.225
‐ Old CVA	30 (13.2)	4 (12.9)	12 (14.8)	14 (12.2)	0.762
Medications					
‐ Antiplatelets	69 (30.4)	4 (12.9)	30 (37.0)	35 (30.4)	0.268
‐ Anticoagulants	26 (11.5)	0 (0)	11 (13.6)	15 (13.0)	0.115
Syncope or dizziness prior head injury	36 (15.9)	0 (0)	8 (9.9)	28 (24.4)	< 0.001
Previous TBI with ICH	13 (5.7)	2 (6.5)	6 (7.4)	5 (4.4)	0.467

At the ED, SBP < 100 mmHg was significantly more common in the unfavorable group (16.1%) compared to the favorable group (2.6%) (*p* = 0.007). Severe initial GCS scores were strongly associated with unfavorable outcomes (51.6%, *p* < 0.001). Pupillary reaction was another potential predictor, with 12.9% of patients in the unfavorable group presenting with nonreactive pupils (*p* < 0.001). Extracranial injuries were observed more frequently in the unfavorable group than in the other groups (*p* = 0.029) (Table 2).

**TABLE 2:** Baseline clinical observations at ED of adult TBI patients with ICH classified by 3 groups of GOS scores.

Variables	All *N* = 227	GOS 1–2 31 (13.6%)	GOS 3–4 81 (35.7%)	GOS 5 115 (50.7%)	*p* for trend
Initial vital signs					
‐ Systolic BP (mmHg) mean ± SD	147.3 ± 31.5	136.3 ± 35.3	150.1 ± 31.9	148.4 ± 29.8	0.172
‐ SBP < 100 mmHg	12 (5.3)	5 (16.1)	4 (4.9)	3 (2.6)	0.007
‐ HR (bpm) mean ± SD	86.4 ± 16.6	97.8 ± 21.0	86.8 ± 17.0	83.1 ± 16.6	< 0.001
‐ RR (per minute) mean ± SD	20.3 ± 2.2	19.6 ± 3.2	20.7 ± 2.4	20.2 ± 1.5	0.650
‐ SpO_2_ (%) mean ± SD	97.3 ± 2.8	96.7 ± 4.3	97.3 ± 2.0	97.4 ± 2.9	0.204
‐ SpO_2_ < 95%	12 (5.3)	4 (12.9)	5 (6.2)	3 (2.6)	0.023
Initial GCS score					
‐ Severe [3–8]	27 (11.9)	16 (51.6)	10 (12.4)	1 (0.9)	< 0.001
‐ Moderate [9–12]	19 (8.4)	8 (25.8)	10 (12.4)	1 (0.9)	
‐ Mild [13–15]	181 (79.7)	7 (22.6)	61 (75.2)	113 (98.2)	
Initial motor response median (IQR)	6 (6, 6)	5 (4, 6)	6 (5, 6)	6 (6, 6)	< 0.001
Pupillary reaction					
‐ One or both nonreaction	5 (2.2)	31 (13.6)	81 (35.7)	115 (50.7)	< 0.001
‐ One or both slight reaction	12 (5.3)	8 (25.8)	4 (5.0)	0 (0)	
‐ Both reaction	210 (92.5)	19 (61.3)	76 (93.8)	115 (100.0)	
Major extracranial injury	44 (19.4)	11 (35.5)	15 (18.5)	18 (15.7)	0.029

CT imaging findings revealed significantly higher rates of SDH (93.6%) and midline shift or brain herniation (64.5%) in the unfavorable group compared to the intermediate and favorable groups (*p* < 0.001). Although most patients received conservative treatment (88.6%), 29.0% of those in the unfavorable group underwent neurosurgical intervention (*p* < 0.001) (Table 3).

**TABLE 3:** CT results, disposition type, and neurosurgical intervention of adult TBI patients with ICH classified by 3 groups of GOS scores.

Variables	All *N* = 227	GOS 1–2 31 (13.6%)	GOS 3–4 81 (35.7%)	GOS 5 115 (50.7%)	*p* for trend
Type of intracranial injuries					
Extra‐axial collection					
‐ EDH	26 (11.5)	8 (25.8)	5 (6.2)	13 (11.3)	0.176
‐ SDH	157 (69.2)	29 (93.6)	63 (77.8)	65 (56.5)	< 0.001
SAH	109 (48.0)	21 (67.7)	38 (46.9)	50 (43.5)	0.034
ICH					
‐ PH	33 (14.5)	6 (19.4)	10 (12.4)	17 (14.8)	0.749
‐ IPH	27 (11.9)	6 (19.4)	14 (17.3)	7 (6.1)	0.009
IVH	17 (7.5)	9 (29.0)	6 (7.4)	2 (1.7)	< 0.001
Midline shift or brain herniation	41 (18.1)	20 (64.5)	14 (17.3)	7 (6.1)	< 0.001
Disposition					
Discharge home and follow‐up	119 (52.4)	7 (22.6)	35 (43.2)	77 (67.0)	< 0.001
General ward	61 (26.9)	7 (22.6)	24 (29.6)	30 (26.0)	
Intensive care unit	46 (20.3)	16 (51.6)	22 (27.2)	8 (7.0)	
Dead at ED	1 (0.4)	1 (3.2)	0 (0)	0 (0)	
Neurosurgical intervention					
Conservative	201 (88.6)	22 (71.0)	70 (86.4)	109 (94.8)	< 0.001
Yes	26 (11.4)	9 (29.0)	11 (13.6)	6 (5.2)	

### 3.2. Factors Associated With Poor 1‐Month GOS Score in Adult TBI Patients With ICH

#### 3.2.1. Univariable Analysis

Univariable analysis identified several significant factors associated with poor 1‐month GOS score groups, including age ≥ 65 years, ambulance transport, high‐energy injury mechanisms, SBP < 100 mmHg, GCS score, abnormal pupillary reaction, SDH, midline shift or herniation, and major extracranial injuries (all *p* < 0.05).

#### 3.2.2. Multivariable Analysis

In the multivariable ordinal logistic regression analysis, several factors were associated with poor 1‐month GOS scores across the favorable, intermediate, and unfavorable groups. Factors associated with poorer outcomes in the model included age ≥ 65 years (mOR 5.25, 95% confidence intervals [CIs] 2.33–11.85), SBP < 100 mmHg (mOR 4.38, 95% CI 1.02–18.86), initial severe (mOR 49.88, 95% CI 14.26–174.44) and moderate (mOR 12.26, 95% CI 3.86–38.98) GCS scores, slight pupillary reaction (mOR 8.36, 95% CI 1.76–39.67), although the estimate was imprecise with wide CIs, SDH (mOR 3.10, 95% CI 1.53–6.25), and midline shift or brain herniation (mOR 4.41, 95% CI 1.84–10.57) (Table 4).

**TABLE 4:** Multivariable ordinal logistic regression analysis of the factors associated with poor 1‐month GOS scores 3 groups (full model and final model).

Variables	mOR	95% CI	*p* value	mOR	95% CI	*p* value
Model	Full			Final (reduced)		
Male	1.10	0.57–2.14	0.778	Not included		
Age ≥ 65 years	9.87	2.91–33.54	< 0.001	5.25	2.33–11.85	< 0.001
Escort by ambulance	0.57	0.20–1.68	0.309	Not included		
High risk mechanism	2.42	0.80–7.26	0.117	Not included		
Underlying diseases						
‐ Neurodegenerative	2.01	0.73–5.51	0.177	Not included		
‐ Old CVA	2.07	0.81–5.26	0.126	Not included		
Medications						
‐ Antiplatelet	0.76	0.36–1.59	0.465	Not included		
‐ Anticoagulants	0.62	0.22–1.73	0.362	Not included		
Previous TBI with ICH	1.93	0.56–6.72	0.300	Not included		
SBP < 100 mmHg	4.13	0.96–17.74	0.056	4.38	1.02–18.86	0.047
SpO_2_ < 95%	0.36	0.07–1.71	0.197	Not included		
Initial GCS score						
‐ Severe [3–8]	58.95	13.12–264.78	< 0.001	49.88	14.26–174.44	< 0.001
‐ Moderate [9–12]	12.48	3.49–44.54	< 0.001	12.26	3.86–38.98	< 0.001
‐ Mild [13–15]	1.00	*p* value = Ref.	1.00	Ref.	Ref.	Ref.
Pupillary reaction						
‐ One or both nonreaction	14.72	0.61–356.34	0.098	11.83	0.47–299.71	0.134
‐ One or both slight reaction	7.62	1.31–44.52	0.024	8.36	1.76–39.67	0.008
‐ Both reaction	1.00	Ref.	Ref.	1.00	Ref.	Ref.
Type of intracranial injuries						
Extra‐axial collection						
‐ EDH	1.36	0.44–4.17	0.595	Not included		
‐ SDH	3.56	1.59–7.99	0.002	3.10	1.53–6.25	0.002
SAH	0.95	0.46–1.97	0.901	Not included		
ICH						
‐ PH	0.98	0.36–2.68	0.970	Not included		
‐ IPH	2.07	0.72–5.91	0.175	Not included		
IVH	2.37	0.65–8.63	0.189	Not included		
Midline shift or brain herniation	4.39	1.72–11.18	0.002	4.41	1.84–10.57	0.001
Major extracranial injury	2.03	0.69–5.98	0.200	Not included		

Neurosurgical intervention was more frequent in the unfavorable group (29.0%) than in the intermediate (13.6%) and favorable (5.2%) groups. This likely reflects greater baseline injury severity among patients selected for surgery rather than an adverse effect of surgery itself.

The overall median ISS was 8 (IQR 5–12), suggesting that many patients did not sustain severe multisystem trauma, although the unfavorable group had substantially higher ISS values.

## 4. Discussion

Consistent with our results, poorer 1‐month GOS outcomes were associated with several factors, including advanced age (≥ 65 years), low initial SBP (SBP < 100 mmHg), initial GCS scores categorized as severe [3–8] or moderate [9–12], slight pupillary reaction, and CT findings such as SDH and midline shift or brain herniation.

Older age was associated with a poorer 1‐month GOS in our cohort [10, 11, 22]. Older patients generally experience poorer outcomes due to decreased physiological resilience and a higher prevalence of comorbidities.

Low initial SBP was associated with poorer 1‐month outcome in our cohort. This finding is biologically plausible because hypotension may worsen secondary brain injury by reducing cerebral perfusion. Current trauma and TBI management guidelines, including the updated ATLS recommendations and recent best‐practice guidelines, emphasize the importance of avoiding hypotension and maintaining adequate cerebral perfusion in patients with TBI [20]. An SBP threshold of 100–110 mmHg is commonly used to define potentially harmful hypotension in TBI. However, this association may partly reflect overall injury severity [20, 21]^.^

Similarly, the association between lower initial GCS and poorer outcome is well established. In our cohort, this relationship remained prominent even when outcomes were evaluated as an ordinal 1‐month functional measure (favorable, intermediate, and unfavorable), suggesting that initial neurological severity remains highly relevant even for early short‐term functional stratification in patients with traumatic ICH [24, 28, 29].

In addition, pupillary reaction is a vital indicator of brainstem function and intracranial pressure. The nonreactive pupils often indicate severe brain injury and elevated intracranial pressure, leading to poor prognoses [6, 28, 30]. In our final model, slight pupillary reaction was associated with poorer outcome, whereas nonreactive pupils were not statistically significant. This finding should be interpreted with caution because the number of patients with abnormal pupillary findings was small, and the CIs were wide, suggesting possible model instability rather than a true absence of prognostic value for nonreactive pupils. This finding should not be interpreted as contradicting established clinical knowledge. Therefore, this variable should be considered exploratory rather than a robust main finding.

Regarding radiological findings, SDH and midline shift or brain herniation was associated with poor 1‐month GOS scores. Imaging findings are crucial in assessing TBI severity. Midline shift or brain herniation was associated with poorer 1‐month GOS in our cohort. However, because midline shift or brain herniation is closely related to overall injury severity and mass effect, this association may partly reflect underlying disease severity [25, 26].

Syncope or dizziness before injury was not associated with a poorer 1‐month outcome and was not retained in the final model. Given the limited number of cases, this finding should be interpreted cautiously. Similarly, antiplatelet and anticoagulant uses were not significantly associated with poorer outcomes in this study. However, this does not exclude a potential effect, as medication‐related risk may depend on factors not captured in our dataset, such as hemorrhage progression, reversal therapy, and overall injury severity. This finding differs from that reported by Powers et al., who found an association with mortality in traumatic ICH [31].

Hypoxia was not significantly associated with poorer 1‐month GOS in this cohort. However, the causes of hypoxia were not systematically analyzed, and hypoxia may have reflected heterogeneous mechanisms such as airway compromise, thoracic trauma, ventilation difficulty, or hemorrhagic shock. Therefore, this finding should be interpreted cautiously [24, 25, 32, 33].

Major extracranial injury was not independently associated with outcome in our final model. This may partly reflect the relatively low overall trauma severity of the cohort, as indicated by the median ISS, rather than the absence of a true clinical relationship. Similarly, Watanabe et al. [29] found that while severe extracranial injuries increase overall morbidity, they do not significantly affect neurological outcomes in TBI patients. However, Perel et al. [34] emphasize that such injuries, particularly those involving severe bleeding, are crucial factors associated with early mortality.

The predominance of mild TBI in this cohort should be considered when interpreting CT‐based associations, because some radiological findings may partly reflect baseline injury severity rather than independent prognostic effects.

These findings may have practical implications for early clinical decision‐making in the ED. From an ED perspective, these findings may support early triage decisions, help determine appropriate levels of monitoring (e.g., ward vs. ICU), and provide preliminary prognostic information to guide discussions with families during the initial phase of care. However, because of the retrospective observational design, the present study does not establish that acting on these factors directly changes the outcome.

Because neurosurgical intervention was not modeled as a time‐dependent treatment variable, its relationship with outcome should be interpreted cautiously, as patients undergoing surgery were likely to have more severe baseline injury.

The use of complete‐case analysis may introduce bias if missingness is not completely random. However, the proportion of missing data in this study was small, and the potential impact on the estimates is likely limited.

A 1‐month outcome is particularly relevant to ED practice because it reflects early recovery trajectories that can inform short‐term planning and decision‐making soon after the acute phase.

### 4.1. Implications for ED Practice

In clinical practice, readily available clinical and imaging variables at ED presentation may help support early risk stratification. This may assist clinicians in prioritizing monitoring intensity, guiding disposition decisions, and facilitating early communication with patients and families regarding short‐term outcomes.

Despite these findings, several limitations should be considered when interpreting the results.

## 5. Limitations

This study has several limitations. First, its retrospective, single‐center design may limit generalizability, and the cohort included a relatively high proportion of older patients. Second, most injuries were blunt trauma, which may not represent other mechanisms of injury. Third, the timing and location of death during follow‐up were not analyzed separately.

Additional limitations include the limited characterization of radiological findings, as lesion volume, timing of CT, and hemorrhagic progression were not systematically recorded. The 1‐month GOS was assessed by different physicians across clinical settings without standardized study‐specific training, which may introduce outcome misclassification. In addition, treatment‐related factors such as neurosurgical intervention were not analyzed as time‐dependent variables. Because lesion severity, volume, and progression were not systematically captured, these findings should be interpreted cautiously. Finally, some regression estimates were imprecise, with wide CIs suggesting possible model instability.

Because GOS assessments were performed by different physicians across clinical settings, interobserver variability may have influenced outcome classification. However, GOS assessment at our institution follows routine clinical practice standards and is commonly used across ED, inpatient, and outpatient settings, which may help maintain a reasonable degree of consistency.

Some estimates were associated with wide CIs, suggesting possible sparse‐data bias and limited precision. Therefore, effect sizes should be interpreted cautiously.

The generalizability of these findings may be limited by local demographic characteristics, trauma patterns (predominantly blunt trauma and elderly patients), and health system factors. These findings may not directly apply to prehospital systems or lower‐resource emergency settings.

## 6. Conclusion

In this retrospective cohort of adult TBI patients with traumatic ICH, older age, low initial SBP, lower GCS scores, SDH, and midline shift were associated with poorer 1‐month GOS scores. These findings may support early risk stratification in the ED. However, they should be interpreted as associations rather than precise effect estimates and do not establish causal relationships or definitive predictions.

Future multicenter studies, particularly those conducted in diverse ED settings, are needed to validate these findings and evaluate whether incorporating early prognostic factors into ED decision‐making improves patient outcomes.

## Funding

No funding was received for this manuscript.

## Ethics Statement

This study received ethical approval from the Committee on Human Rights Related to Research Involving Human Subjects at the Faculty of Medicine, Mahidol University’s Ramathibodi Hospital, on September 22, 2023 (IRB COA. MURA2023/728). Due to its retrospective design, the need for informed consent from individual patients was waived.

## Consent

Written informed consent was waived due to the retrospective study design, in accordance with ethical principles for research involving human subjects as outlined in the Declaration of Helsinki.

## Conflicts of Interest

The authors declare no conflicts of interest.

## Data Availability

The data that support the findings of this study are available on request from the corresponding author. The data are not publicly available due to privacy or ethical restrictions.
